# Supplementary material for: Lanthanum modulated reaction pacemakers on a single catalytic nanoparticle
Source: Nat Commun. 2023 Nov 8;14:7186. doi: 10.1038/s41467-023-43026-3 (PMC10632447; doi:10.1038/s41467-023-43026-3)
Supplement: Supplementary file 1 — Supplementary Information [file 41467_2023_43026_MOESM1_ESM.pdf]

Lanthanum modulated reaction pacemakers on a single catalytic nanoparticle

Maximilian Raab[1], Johannes Zeininger[1], Yuri Suchorski[1], Alexander Genest[1], Carla Weigl[1], Günther Rupprechter[1]*

[1]*Institute of Materials Chemistry, TU Wien; Getreidemarkt 9, 1060 Vienna, Austria*

Supplementary Information

Supplementary Note 1: Determination of the La coverage

The image intensity in FEM is strongly dependent on the work function of the sample. This allows detection and monitoring of species adsorbed on the tip surface which cause observable changes in the surface work function. For most adsorbates, particularly for electropositive adsorbates such as alkali, alkali earth and rare earth metals, the work function unambiguously depends on their surface coverage. This enables the determination of the adsorbate coverage, provided the coverage dependence of the work function is known. This approach is not limited to FEM and has often been used for the coverage calibration in surface studies[1–3]. In the current investigation, the coverage dependence of the work function in the La/Rh adsorption system was employed to quantify the coverage of La used as a promotor in H_2 oxidation on a Rh nanotip.

For calibration of the coverage, La was evaporated at room temperature onto a Rh-nanotip from a miniature home-made Knudsen-type effusion cell serving as La-evaporator. After each evaporation dosage, the La-layer was annealed at 550 K to equilibrate the La-coverage across the sample surface via surface diffusion. After equilibration, FEM images were taken and the imaging voltage was adjusted to maintain constant image intensity (constant emission current). Using field desorption[4,5] the La-layer could be fully removed from the Rh-nanotip surface and an atomically clean Rh surface could be restored at any time.

The work function of the La covered surface was estimated using the following equation[6,7]

$$\varphi_{\mathrm{La}} = \varphi_{\mathrm{Rh}} \left(\frac{U_{\mathrm{La}}}{U_{\mathrm{Rh}}}\right)_{I\,=\,\mathrm{const}}^{2/3} \qquad \textbf{(1.1)}$$

where φ_{La} is the work function of the La-covered surface and φ_{Rh} is the work function of the clean Rh surface, U_{La} and U_{Rh} are the corresponding imaging voltages necessary to keep constant field emission current *I*.

The work function of low Miller index Rh surfaces ranges from 4.9 eV to 5.4 eV[8], while for polycrystalline Rh an average work function of 4.9 eV was determined[9]. As our tip consists of many nanofacets of different crystallographic orientations, this value was adopted for our estimations. However, it should be noted that not the absolute value of the work function, but its adsorption caused variation is meaningful in the present case.

Supplementary Fig. 1 depicts the measured dependence of the work function on the La-evaporation time. The curve reflects the known properties of electropositive adsorbates on transition metal surfaces[10,11]: an initial sharp linear decay of the work function related to dipole formation at low coverages and a slow flattening of the curve due to proceeding depolarization[12] caused by dipole-dipole interaction[13,14] at increasing coverage. After a weakly pronounced minimum, the curve reaches a plateau which is associated with the formation of a monolayer[10,11].

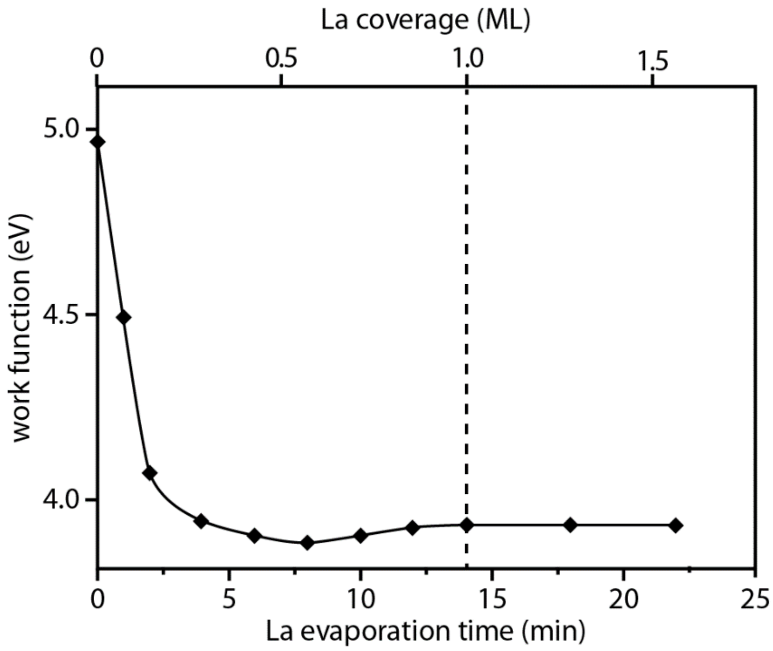

Supplementary Fig. 1. Dependence of the work function of the La-covered Rh-nanotip surface on the La-evaporation time.

The onset of the plateau formation at 14 min is attributed to 1 ML of La and was used for La-coverage estimation in the present catalytic experiments. The initial linear decrease of the work function for small coverages and exponential dependence of the emission current (image intensity) on the work function[15] facilitate the La-coverage estimation at submonolayer coverages used in the present study.

The adsorption of La on Rh has, to our knowledge, not been studied yet, but the obtained curve strongly resembles analogue curves for La on W[16] or Mo[17]. The calibration of the coverage of electropositive adsorbates on transition metal surfaces based on the formation of a monolayer was repeatedly confirmed by independent LEED studies[16–18] and has thus been routinely applied for coverage determination in diffusion[1,2,19,20], other surface physics experiments[3,21] and even as a physical basis for coverage-calibration devices[2].

Verifying the work function after each experiment indicated that the La-coverage on the imaged surface had not remarkably changed. The hours-long stability of self-sustained periodic oscillations at constant external parameters additionally confirmed the constancy of the La-coverage in catalytic experiments.

Supplementary Note 2: Transition point tracking

Transition point tracking (TPT) allows determining the reaction pacemakers based on the pixel-wise determination of the time points of the local kinetic transitions from the measured local timeseries of a reaction front propagation. From these calculated local transition times, the propagation of the reaction front can be reconstructed, as the reaction front is a travelling continuum of local kinetic transition points. The concept is illustrated in Supplementary Fig. 1.

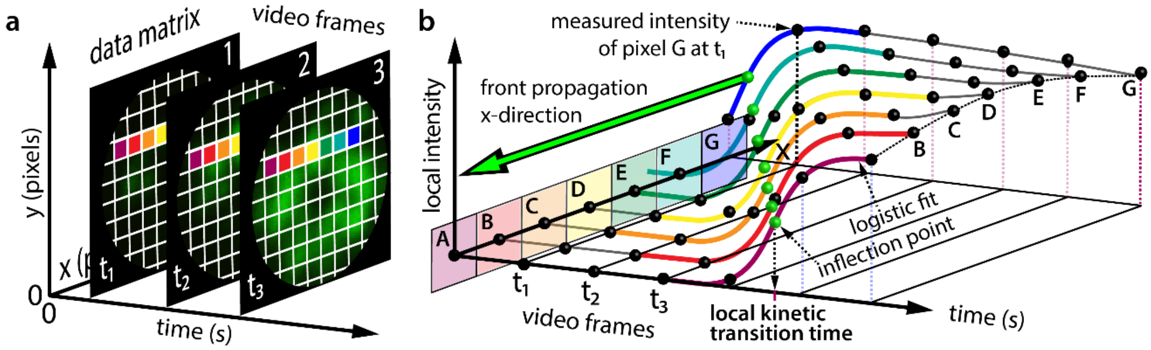

Supplementary Fig. 2. Transition Point Tracking (TPT): (**a**) the FEM video contains local time series data of individual pixel intensities; (**b**) The discrete intensity timeseries measured for each pixel can be converted into continuous "true time" curves by a logistic fit. The local kinetic transition times (green dots) are then determined by the inflection points of the "true time" dependence reconstructed by the logistic fit.

For each pixel, the local FEM intensity is extracted from the FEM video file (Supplementary Fig. 1a) and evaluated. The local transition points, both τ_A and τ_B, are determined as the inflection points of the time-dependent local FEM intensity (Supplementary Fig. 2b). The exact assignment of the inflection point requires a reconstruction of the continuous image intensity progression from the measured discrete data points, which is achieved via fitting the timeseries, assuming an underlying sigmoidal time dependence of the reaction rate. This assumption is based on the Langmuir-Hinshelwood mechanism of the investigated hydrogen oxidation reaction[22] and considers that initially, due to the autocatalytic behaviour of the kinetic transition to the active state, an exponential growth in the reaction rate is observed[23,24]. Limited by the total available adsorption sites, the increase of intensity eventually levels off, and the intensity growth converges to its maximum. This behaviour is characteristic for logistic growth; therefore, a sigmoidal fit is appropriate. The local transition time is then determined from the inflection point of the fit function, calculated from the maximum of its derivative. Plotting the determined transition times for each pixel using a colour-coded timescale generates a "raw" transition map which summarises the propagation of a distinct reaction front. However, diffusion and reaction-induced fluctuations accompanying the reaction[25–27] cause small uncertainties in the exact transition times, leading to imperfections in the calculated "raw" transition map. Therefore, post-processing in the form of a region-based smoothing algorithm is applied: the transition time of any pixel is determined by also taking the "raw" transition time values of surrounding pixels within a certain distance into consideration. A distribution analysis is performed of all the local "raw" transition times within this distance. Outliers caused by fluctuations are then removed by only using the main peak of the distributions' histogram. From the remaining values, the new transition time (τ) is determined by weighted averaging

$$\tau = \frac{\sum_{i=1}^{N} \tau_{raw,i} \cdot \varepsilon_i \cdot w_i}{\sum_{i=1}^{N} \varepsilon_i \cdot w_i}, \quad \textbf{(2.1)}$$

where N is the number of pixels within the chosen distance and $\tau_{\mathrm{raw},i}$ is the "raw" local transition time. The parameter ε_i is 1 if the respective "raw" transition time is included in the main peak of the distribution histogram and 0 if it is an outlier. The weight w_i of the value for each surrounding pixel is defined using a Gauss relation based on its distance to the local centre pixel. This processing ensures a high accuracy of the generated transition maps, which allows exact locating of the pacemakers and visualizing the local front propagation.

On the one hand, TPT allows the detection of the pacemaker responsible for the nucleation of the evaluated reaction front by retracing the point with the lowest transition time[28]. The local transition time can then also be displayed relative to the time of front nucleation, which gives insight into the time the front needs to reach certain regions. On the other hand, the transition maps illustrate exactly how far the front is spreading and where the front is slowed down or even stopped, thereby providing information about diffusion barriers limiting the interfacet coupling and compartmentalisation of the reaction. Due to the sigmoidal reconstruction being a continuous function, the determined transition time point can also lie between two frames that follow one another, thereby providing a much-improved time resolution than the original images. An essential advantage of the TPT method is that it is purely based on the relative increase of the local FEM intensity and the shape of its progression, so pixels with intensities on the lower end of the dynamic imaging range are treated in exactly the same way as those with high image intensity.

Supplementary Note 3: The role of the electric field

In a field emission microscope (FEM) the surface of a nm-sized hemispherical apex of a tip-shaped specimen is imaged by electrons field emitted via tunnelling into vacuum due to an applied electrostatic field of 3 to 5 V/nm[29]. Therefore, possible field effects on the studied phenomena should be considered.

Since kinetics of catalytic H_2 oxidation is governed by energetics of hydrogen and oxygen adsorption, the possible field effect must result from the field dependence of the binding energies of O and H atoms. The physical origin for such a dependence lies in the field induced redistribution of electron density near the metal surface[30], which was the subject of previous experimental and theoretical studies, recapitulated in a review[31].

Such a field induced redistribution of surface electron density may indeed modify the binding energy of adsorbates, as directly measured on an atomic scale by field ion appearance energy spectroscopy (FIAES) for CO, O_2, N_2 and other adsorbates, as summarized in a review[32]. The impact of such effect on the reaction kinetics can also be directly measured, e.g., for CO oxidation on Pt[33].

The above effect on the binding energy of adsorbates takes place, however, only at applied field strengths significantly above ~10 V/nm. This limits the possible field-effect, since only a "positive" field of such strength, i.e., a field directed away from the surface (positively charged sample surface) and "pushing" electrons into the bulk as, e.g., in a field ion microscope (FIM), can be applied to a metal surface. A "negative" electric field (i.e., of the direction used in an FEM) of field strengths necessary for modification of binding energy of adsorbates would lead to avalanche like field emission, resulting eventually in explosive electron emission and destruction of the surface[34]. This limits the field strengths applied in a FEM to values, at which the field-induced electron density redistribution and thus field effects on the binding energy of adsorbates such as oxygen, hydrogen, or carbon monoxide do not take place. Consequently, this excludes field effects on the kinetics of H_2 oxidation in the FEM conditions, as previously noticed in Ref.[35] and discussed in detail in Ref.[36].

Supplementary Note 4: Micro-kinetic model simulations

The micro-kinetic model is based on the Langmuir-Hinshelwood reaction mechanism with formation and depletion of subsurface oxygen as the feedback mechanism governing the oscillations. Both, the reaction mechanism and the subsurface oxygen feedback mechanism are well established for oscillating hydrogen oxidation on Rh[37–41].

The oscillation mechanism is summarized in Supplementary Fig. 3a. Both reactants, hydrogen and oxygen, adsorb dissociatively to the surface from the gas phase. The adsorption of hydrogen and oxygen is competitive, where the "winner" of the adsorption process is governed by the temperature and the respective partial pressures of the gases. Under the applied experimental conditions, oxygen adsorbs via a molecular precursor (Supplementary Fig. 3a A_1) with a following dissociation into two chemisorbed oxygen atoms (Supplementary Fig. 3a A_2) for each molecule, covering the clean Rh surface. In this oxygen-covered state, the dissociative adsorption of hydrogen (Supplementary Fig. 3a A_3), which requires two free adjacent adsorption sites, is blocked and the reaction is in a catalytically inactive state. In the inactive state, oxygen can now migrate into subsurface positions by diffusion (Supplementary Fig. 3a D_1), which occurs preferentially at low coordinated atoms, where the activation energy for this process is lowest. The presence of subsurface oxygen reduces the sticking coefficient of oxygen. This lowers the oxygen adsorption rate and leads to a favoured dissociative adsorption of hydrogen. The adsorbing hydrogen reacts with the adsorbed oxygen, forming H_2O (Supplementary Fig. 3a R), which desorbs immediately at reaction conditions above 400 K[42,43]. The reaction thereby creates free adsorption sites which are preferentially filled by hydrogen adsorbing from the gas phase and removes the dense adsorbed oxygen layer. The reaction turns thus to the catalytically active state, where both hydrogen and oxygen can adsorb and form water. Due to the removal of the dense surface oxygen layer, the subsurface oxygen atoms are driven back to the surface by the gradient of the chemical potential, where they are consumed by the reaction with hydrogen. Emptying of the subsurface oxygen depot restores the sticking coefficient of oxygen to a level sufficient for the preferential adsorption of oxygen. Thereby, the reaction switches back to the inactive state and the oscillation cycle is closed.

The model, originally introduced by McEwen et al.[44,45] and based on the above general considerations, was first used to simulate field-induced oscillations observed using FIM[46]. It therefore contained terms describing the reduction of the activation energy barrier for subsurface oxygen formation by a high electric field of > 10 V/nm. In case of FEM, used in the present work. however, field-effects can be neglected (see Supplementary Note 3), thus the field-dependent terms were not considered in the present modelling.

The original model was a "single oscillator" model. As the communication between adjacent surface regions plays an important role in spatio-temporal reaction processes, the current model was developed as a system of coupled oscillators of five different types: two different oscillators representing the respective experimentally found pacemaker regions (called A and B in Fig. 5a), as well as the three types of low-Miller-index facets. The choice of the model network is based on the crystallographic layout and symmetry of the present nanocrystal surface (see Fig. 5a).

Coupling between adjacent surface regions via surface diffusion of hydrogen (Supplementary Fig. 3b) was introduced by corresponding diffusion terms[36,47]. Oxygen diffusion is three orders of magnitude slower than that of oxygen under the present conditions and was therefore neglected[44,45].

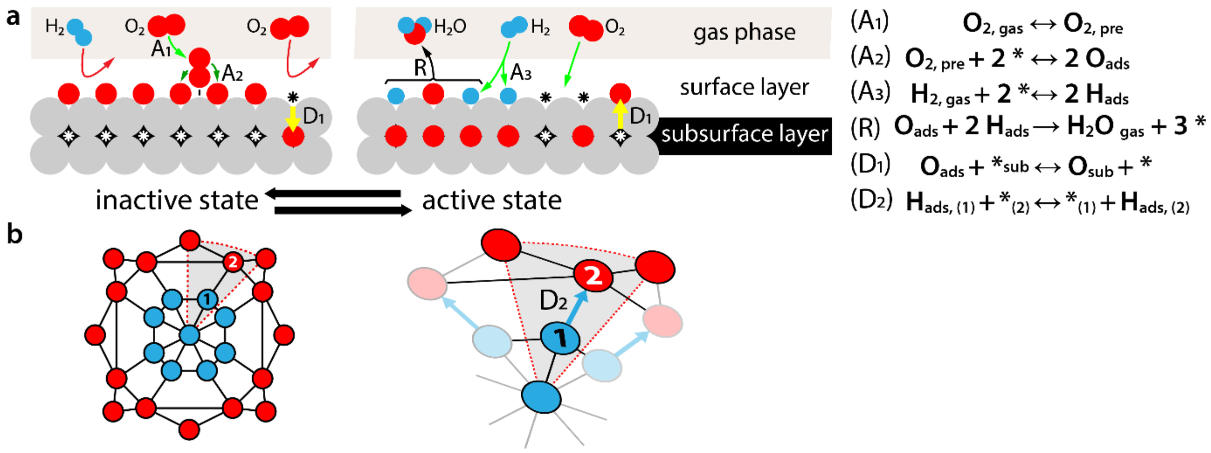

Supplementary Fig. 3. Schematic illustration of the oscillation mechanism: **(a)** profile view on local reaction kinetics showing active and inactive state (A_1 to R). Formation/Depletion of subsurface oxygen (D_1) acts as feedback mechanism. Colour-code: Rh (grey), O (red), and H (blue). (*) represents empty sites; **(b)** hydrogen diffusion (D_2) provides lateral coupling between different oscillators.

In the coupled-oscillator model, the hydrogen coverage θ_H^i, the oxygen coverage θ_O^i and the subsurface coverage θ_S^i are described by the following kinetic equations:

$$\frac{d\theta_H^i}{dt} = 2k_a^H p_{H_2} {\theta_*^i}^2 - 2k_d^H {\theta_H^i}^2 - 2k_r^i \theta_H^i \theta_O^i + k_{dif}^H \sum_{j\neq i}^{n} C_{i,j}\left(\theta_H^j \theta_*^i - \theta_H^i \theta_*^j\right), \qquad \textbf{(4.1)}$$

$$\frac{d\theta_O^i}{dt} = \frac{2}{1+K^i{\theta_*^i}^2}\left(k_a^O K^i p_{O_2} {\theta_*^i}^2 - k_d^{O,i} {\theta_O^i}^2\right) - k_{ox}\theta_O^i\left(1-\theta_S^i\right) + k_{red}^i \theta_S^i \theta_*^i - k_r^i \theta_H^i \theta_O^i, \qquad \textbf{(4.2)}$$

$$\frac{d\theta_S^i}{dt} = k_{ox}\theta_O^i\left(1-\theta_S^i\right) - k_{red}^i \theta_S^i \theta_*^i, \qquad \textbf{(4.3)}$$

where i is the index of the respective oscillator, and θ_*^i represents the empty surface sites defined as $\theta_*^i = 1 - \theta_H^i - \theta_O^i$. The rate and coupling constants are given by

$$k_a^H = S_0^H\, a_s / \sqrt{2\pi m_{H_2}/\beta} \qquad \textbf{(4.4)}$$

$$k_d^H = k_{d0}^H e^{-\beta E_d^H} \qquad \textbf{(4.5)}$$

$$k_r^i = k_r^0 e^{-\beta\left(E_r + A_r^H \theta_H^i + A_r^O \theta_O^i\right)} \qquad \textbf{(4.6)}$$

$$k_{dif}^{H} = k_{dif0}^{H} e^{-\beta E_{dif}^{H}} \quad \textbf{(4.7)}$$

$$C_{i,j} = w_{i,j}/{d_{i,j}}^2 \quad \textbf{(4.8)}$$

$$K^i = K_0 e^{-\beta\left(E_K + A_K^O\, \theta_O^i + A_K^S \theta_S^i\right)} \quad \textbf{(4.9)}$$

$$k_a^O = S_0^O\, a_s / \sqrt{2\pi m_{O_2} k_B T} \quad \textbf{(4.10)}$$

$$k_d^{O,i} = k_{d0}^O e^{-\beta\left(E_d^O + A_d^O\, \theta_O^i + B_d^O\, {\theta_O^i}^2\right)} \quad \textbf{(4.11)}$$

$$k_{ox} = k_{ox}^0 e^{-\beta E_{ox}^i} \quad \textbf{(4.12)}$$

$$k_{red}^i = k_{red}^0 e^{-\beta\left(E_{red}^i + A_{red}^S \theta_S^i\right)}, \quad \textbf{(4.13)}$$

where S_0^H and S_0^O correspond to the initial sticking coefficients of hydrogen and oxygen, a_s is the area of a surface site of 10 Å^2, m_{H_2} and m_{O_2} represent the average molecular masses of hydrogen and oxygen, while $\beta = 1/k_B T$.

The local natural frequency of the oscillating reaction on a certain surface region depends on the rate of subsurface oxygen formation/depletion, which is largely determined by the activation energy E_{ox} for the formation of subsurface oxygen[28,38–41] and thereby is related to the surface roughness, i.e., to the crystallographic orientation of the Rh surface[48]. The E_{ox}^i values were assigned to each oscillator of the model network, whereby the “flat” low-Miller-index surfaces have higher activation energies for subsurface oxygen formation. The B region oscillator (see Fig. 5a), representing the “fast” pacemaker of the experimentally observed oscillations, was accordingly assumed to have a low E_{ox}^i, while the A site, representing the slower oscillations, was assigned a comparably higher E_{ox}^i. As the local atomic roughness of the surface strongly depends on the curvature of the crystal, the used values should be treated as effective E_{ox} values, which generally are lower than those of planar macroscopic single crystals. The effective activation energy for subsurface oxygen reduction E_{red}^i was directly inferred by the relation[39]

$$E_{red}^i = 0.293 + 0.776\, E_{ox}^i\,. \quad \textbf{(4.14)}$$

The E_{ox}^i and the E_{red}^i values used for the different oscillator types, as well as the locally assumed oxygen sticking coefficients, are listed in Supplementary Table 1.

For the La-precovered Rh surface, the modified adsorption behaviour of oxygen due to its enhanced binding energy was considered. Accordingly, the activation energy for oxygen desorption (E_d^O) and the oxygen sticking coefficients were adjusted. Additionally, the coverage

dependence of adsorbed oxygen (A_K^O) and of subsurface oxygen (A_K^s) on oxygen dissociation were modified. The parameters that were changed for representing the La/Rh systems are listed in Supplementary Table 2. The effect of coadsorbed La on the subsurface oxygen formation was considered by adjusting the local E_{ox}^i values. Additionally, the La can also cover favorable sites for subsurface oxygen incorporation, i.e., at step/kink sites which are mostly present at type B sites. Therefore, the E_{ox}^i for these oscillators B, was increased more than the other E_{ox}^i values. The respective values for the La/Rh system are also listed in Supplementary Table 1.

Supplementary Table 1. Local effective activation energies for subsurface oxygen formation (E_{ox}^i) and reduction (E_{red}^i) for the Rh and the La/Rh systems and local initial oxygen sticking coefficients (S_0^O). Energies are given in eV.

class	E_{ox}^i(Rh)	E_{red}^i(Rh)	S_0^O(Rh)	E_{ox}^i(La/Rh)	E_{red}^i(La/Rh)	S_0^O(La/Rh)
A	0.87	0.97	0.620	0.90	0.99	0.640
B	0.65	0.80	0.620	0.85	0.95	0.655
{100}	0.94	1.02	0.620	0.99	1.06	0.640
{110}	0.89	0.98	0.720	0.94	1.02	0.740
{111}	0.91	1.00	0.770	0.96	1.04	0.790

The diffusion flux between adjacent surface regions is proportional to $C_{i,j}$, which represents the distance-weighted coupling coefficient between oscillators i and j, which depends on $d_{i,j}$, the distance between the respective oscillators, which was based on the size of the tip apex with a radius of 18 nm. The $w_{i,j}$ values are coupling factors which are all set to 1 in the present calculations. The kinetic parameters used in this study and listed in Supplementary Table 2 are based on our previous coupled-oscillator model[47], with an adjustment due to the smaller sample size used in the present work. The stability of the calculated results to small changes of kinetic parameters was ensured by routinely performed extensive testing. The La-modified oscillations are stable for a range of modified oxygen adsorption parameters, i.e., for E_d^O between 3.2 – 5.0 eV, A_K^s between 0.070 – 0.145 eV and A_K^O between 0.095 – 0.145 eV. The parameters in Supplementary Table 2 reproduce best the experimentally observed oscillation frequencies.

Supplementary Table 2. Kinetic parameters used in the micro-kinetic model simulations. The values that were changed to represent the La-precovered Rh surface are additionally listed. Energies are given in eV, rate constants in 1/s and k_{dif0}^{h} in nm²/s.

symbol	parameter description	Rh	La/Rh
S_0^H	initial sticking coefficient of H	0.3	0.3
k_{d0}^{h}	pre-factor for hydrogen desorption	3.0 x 10^{10}	3.0 x 10^{10}
E_d^H	desorption energy of H	0.6	0.6
k_r^0	pre-factor for water formation	7.0 x 10^{12}	7.0 x 10^{12}
E_r	activation energy for water formation	0.79	0.79
A_r^H	coverage dependence of the activation energy of water formation on H	-0.27	-0.27
A_r^O	Coverage dependence of the activation energy of water formation on O	-0.145	-0.145
k_{dif0}^{h}	pre-factor for hydrogen diffusion	1.5 x 10^{6}	1.5 x 10^{6}
E_{dif}^{H}	activation energy for hydrogen diffusion	0.187	0.187
K_0	pre-factor for the oxygen dissociation equilibrium constant	0.2525	0.2525
E_k	activation energy for the oxygen dissociation equilibrium constant	-0.178	-0.178
A_K^O	coverage dependence of adsorbed oxygen on oxygen dissociation	0.158	0.125
A_K^s	coverage dependence of subsurface oxygen on oxygen dissociation	0.0558	0.108
k_{d0}^{O}	pre-factor for oxygen desorption	6.0 x 10^{13}	6.0 x 10^{13}
E_d^O	desorption energy of O	2.85	4.00
A_d^O	coverage dependence of the oxygen desorption energy on adsorbed oxygen	-0.4	-0.4
B_d^O	Coverage dependence of the oxygen desorption energy on molecular oxygen	-0.5	-0.5
k_{ox}^{0}	pre-factor for oxygen diffusion from surface to subsurface sites	9.05 x 10^{6}	9.05 x 10^{6}
k_{red}^{0}	pre-factor for oxygen diffusion from subsurface to surface sites	7.90 x 10^{8}	7.90 x 10^{8}
A_{red}^{s}	subsurface oxygen coverage dependence on surface-subsurface reduction	0.26	0.26

Supplementary Note 5: Density Functional Theory calculations

To evaluate how co-adsorbed lanthanum modify the oxygen binding energy on Rh, density functional theory calculations were carried out, using the VASP program package (version 6.4.1[49,50]) with the PBE functional[51] employing the projector augmented wave formalism and a cutoff energy of 400 eV[52,53]. A 4x4 3-layer Rh(111) slab was used, where the "top" layer was allowed to fully relax, whereas the "bottom" layers were constrained to the calculated Rh bulk distance of 269 pm. Each layer contains 16 Rh atoms, so that the whole slab model consists of 48 Rh atoms. A vacuum layer of 1000 pm was added in z-direction and a k-point grid of 3x3x1 was used. Geometry optimizations were considered converged when the forces on atoms dropped below 0.0002 eV/pm. Binding energies of n oxygen atoms are reported as the average binding energy of all n O moieties at the surface, referenced to each transfer of half of an oxygen molecule from the gas phase to the surface, according to the following equation: Rh(111) +n/2 O_2(g) → n×O@Rh(111). Therefore, negative binding energies correspond to a favorable interaction of the oxygen atom(s) with the surface. Note that typical DFT calculations reproduce energies in a range of ~0.1 to 0.2 eV, but trends can still be captured well[54,55].

In a low coverage situation of 1/16, the binding energy of an oxygen atom at an fcc position of the bare Rh(111) surface was -2.08 eV, well within the range of previous reports at ¼ coverage of ~1.73 eV (rPBE)[56] and 2.24 eV (PW91)[57] (Supplementary Figure 4a). When a lanthanum atom is present, the oxygen binds directly at the interface between Rh and La, yielding a binding energy of -2.11 eV (Supplementary Fig. 4b). In contrast, an oxygen further away from La has a reduced binding energy of -2.05 eV (not shown). Interestingly, two oxygen atoms increase the average binding energy at a La-Rh site to -2.59 eV (Supplementary Fig. 4c), whereas on the bare Rh surface higher oxygen coverages lead to smaller binding O energies[58]. Adding a third oxygen atom at a La-Rh site yields an average binding energy of -2.61 eV (Supplementary Fig. 4d), much stronger than oxygen at 1/16 coverage at the bare Rh(111) surface. A fourth oxygen in the vicinity of La leads to a drop in average binding energy to -2.45 eV, indicating the beginning of saturation effects. Overall, these exploratory density functional calculations indicate that already the presence of a single La adatom in low coverage of 1/16 significantly affects the binding energy of several oxygen atoms in the neighborhood, This, in turn, affects subsequent reaction steps.

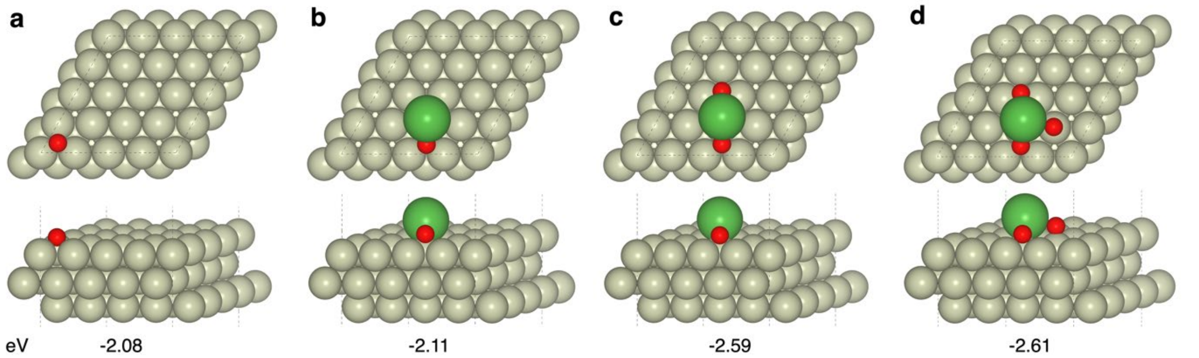

Supplementary Fig. 4. Adsorption structures of oxygen and lanthanum at a Rh(111) surface, upper panels - top views, bottom panels - side views. The values below are the averaged oxygen binding energies per O moiety in eV, with molecular oxygen in the gas phase as reference. Grey spheres represent Rh, red spheres oxygen and green spheres lanthanum: (**a**) single O atom without La; (**b**) La and a single O atom; (**c**) La and two O atoms; (**d**) La and three O atoms.

Supplementary References:

1. Naumovets, A. G., Paliy, M. V. & Vedula, Yu. S. Spreading of a rarefied phase and nonmonotonic concentration profiles in surface diffusion of lithium on the (112) plane of molybdenum. *Phys. Rev. Lett.* **71**, 105–108 (1993).
2. Suchorski, J. S. Surface diffusion of potassium on (100) and (111) germanium planes. *Surf. Sci.* **231**, 130–134 (1990).
3. Suchorski, Y. & Hupalo, M. S. Coadsorption of lithium and oxygen on W(112): Nanosized facets versus single crystals. *Ultramicroscopy* **111**, 381–385 (2011).
4. Müller, E. W. Field Desorption. *Phys. Rev.* **102**, 618–624 (1956).
5. Suchorski, Yu. *et al.* Field desorption and field evaporation of metals. *Prog. Surf. Sci.* **53** 135–153 (1996).
6. Błaszczyszyn, R., Błaszczyszyn, M. & Męclewski, R. Work function of the adsorption system of potassium on tungsten. *Surf. Sci.* **51**, 396–408 (1975).
7. Błaszczyszyn, M. Work function of adsorption systems potassium on tantalum and on molybdenum. *Surf. Sci.* **59**, 533–540 (1976).
8. Methfessel, M., Hennig, D. & Scheffler, M. Trends of the surface relaxations, surface energies, and work functions of the 4 *d* transition metals. *Phys. Rev. B* **46**, 4816–4829 (1992).
9. Michaelson, H. B. The work function of the elements and its periodicity. *J. Appl. Phys.* **48**, 4729–4733 (1977).
10. Bol'shov, L. A., Napartovich, A. P., Naumovets, A. G. & Fedorus, A. G. Submonolayer films on the surface of metals. *Sov. Phys. Uspekhi* **20**, 432–451 (1977).
11. Naumovets, Anton. G. Adsorption of Alkali and Other Electropositive Metals. in *Surface and Interface Science - Volume 5: Solid-Gas Interfaces I* (ed. Wandelt, K.) 157–206 (Wiley, 2016). doi:10.1002/9783527680573.ch34.
12. Verhoef, R. W. & Asscher, M. The work function of adsorbed alkalis on metals revisited: a coverage-dependent polarizability approach. *Surf. Sci.* **391**, 11–18 (1997).
13. Braun, O. M. & Medvedev, V. K. Interaction between particles adsorbed on metal surfaces. *Sov. Phys. Uspekhi* **32**, 328–348 (1989).
14. Vedula, Yu. S., Loburets, A. T., Lyuksyutov, I. F., Naumovets, A. G. & Poplavskij, V. V. Surface diffusion and interaction of adsorbed particles of electropositive elements on refractory metals. *Kinet. Catal.* **31**, 270–288 (1990).
15. Good, R. H. & Müller, E. W. Field Emission. in *Electron-Emission Gas Discharges I / Elektronen-Emission Gasentladungen I* vol. 4 / 21 176–231 (Springer Berlin Heidelberg, 1956). doi: 10.1007/978-3-642-45844-6_2
16. Losovyj, Ya. B. *et al.* Adsorbtsiya bariya i lantana na grani (111) kristalla vol'frama. *Fiz. Tverd. Tela* **28**, 3693–3698 (1968).

17. Losovyj, Ya. B., Medvedev, V. K., Smereka, T. P., Palyukh, B. M. & Babkin, G. V. Adsorption of lanthanum on a (112) face of a molybdenum crystal. *Sov. Phys. Solid State* **24**, 1213–1216 (1982).

18. Fedorus, A. G., Naumovets, A. G. & Vedula, Yu. S. Adsorbed barium films on tungsten and molybdenum (011) face. *Phys. Status Solidi A* **13**, 445–456 (1972).

19. Naumovets, A. & Vedula, Y. Surface diffusion of adsorbates. *Surf. Sci. Rep.* **4**, 365–434 (1985).

20. Naumovets, A. G., Paliy, M. V., Vedula, Yu. S., Loburets, A. T. & Senenko, N. B. Diffusion of lithium and strontium on Mo(112). *Prog. Surf. Sci.* **48**, 59–70 (1995).

21. Naumovets, A. G. Adsorption on metals: a look from the not-too-far East. *Surf. Sci.* **299–300**, 706–721 (1994).

22. Zum Mallen, M. P., Williams, W. R. & Schmidt, L. D. Steps in hydrogen oxidation on rhodium: hydroxyl desorption at high temperatures. *J. Phys. Chem.* **97**, 625–632 (1993).

23. Africh, C. *et al.* Water Production Reaction on Rh(110). *J. Am. Chem. Soc.* **127**, 11454–11459 (2005).

24. Africh, C. & Comelli, G. Scanning tunnelling microscopy investigations of simple surface reactions on Rh(110). *J. Phys.: Condens. Matter* **18**, R387–R416 (2006).

25. Suchorski, Yu., Beben, J., James, E. W., Evans, J. W. & Imbihl, R. Fluctuation-Induced Transitions in a Bistable Surface Reaction: Catalytic CO Oxidation on a Pt Field Emitter Tip. *Phys. Rev. Lett.* **82**, 1907–1910 (1999).

26. Suchorski, Yu. *et al.* Fluctuations and critical phenomena in catalytic CO oxidation on nanoscale Pt facets. *Phys. Rev. B* **63**, 165417 (2001).

27. Suchorski, Y. Surface Diffusion Via Adsorbate Density Fluctuations. in *Encyclopedia of Interfacial Chemistry* 648–665 (Elsevier, 2018). doi:10.1016/B978-0-12-409547-2.12943-9.

28. Zeininger, J. *et al.* Single-Particle Catalysis: Revealing Intraparticle Pacemakers in Catalytic H_2 Oxidation on Rh. *ACS Catal.* **11**, 10020–10027 (2021).

29. Good, R. H. & Müller, E. W. Field Emission. in *Electron-Emission Gas Discharges I / Elektronen-Emission Gasentladungen I* vol. 4 / 21 176–231 (Springer Berlin Heidelberg, 1956).

30. Kostrobiy, P. P., Markovych, B. M. & Suchorski, Y. Revisiting Local Electric Fields on Close-Packed Metal Surfaces: Theory Versus Experiments. *SSP* **128**, 219–224 (2007).

31. Suchorski, Yu., Schmidt, W. A., Ernst, N., Block, J. H. & Kreuzer, H. J. Electrostatic fields above individual atoms. *Prog. Surf. Sci.* **48**, 121–134 (1995).

32. Suchorski, Y. Probing adsorption on a nanoscale: field desorption microspectroscopy. *Adsorption* **23**, 217–224 (2017).

33. Suchorski, Y., Imbihl, R. & Medvedev, V. K. Compatibility of field emitter studies of oscillating surface reactions with single crystal measurements: catalytic CO oxidation on Pt. *Surf. Sci.* **401**, 392–399 (1998).

34. Litvinov, E. A., Mesyats, G. A. & Proskurovskiĭ, D. I. Field emission and explosive electron emission processes in vacuum discharges. *Sov. Phys. Usp.* **26**, 138–159 (1983).

35. Datler, M. *et al.* Hydrogen Oxidation on Stepped Rh Surfaces: µm-Scale versus Nanoscale. *Catal. Lett.* **146**, 1867–1874 (2016).

36. Raab, M., Zeininger, J., Suchorski, Y., Tokuda, K. & Rupprechter, G. Emergence of chaos in a compartmentalized catalytic reaction nanosystem. *Nat. Commun.* **14**, 736 (2023).

37. Yates, J. T., Thiel, P. A. & Weinberg, W. H. The catalytic reaction between adsorbed oxygen and hydrogen on Rh(111). *Surf. Sci.* **82**, 45–68 (1979).

38. Suchorski, Y. *et al.* Visualizing catalyst heterogeneity by a multifrequential oscillating reaction. *Nat. Commun.* **9**, 600 (2018).

39. Suchorski, Y. *et al.* Surface-Structure Libraries: Multifrequential Oscillations in Catalytic Hydrogen Oxidation on Rhodium. *J. Phys. Chem. C* **123**, 4217–4227 (2019).

40. Suchorski, Y. *et al.* Resolving multifrequential oscillations and nanoscale interfacet communication in single-particle catalysis. *Science* **372**, 1314–1318 (2021).

41. Winkler, P. *et al.* Coexisting multi-states in catalytic hydrogen oxidation on rhodium. *Nat. Commun.* **12**, 6517 (2021).

42. Gregoratti, L. *et al.* Structural effects on water formation from coadsorbed H + O on Rh(100). *Surf. Sci.* **340**, 205–214 (1995).

43. Verheij, L. K. & Hugenschmidt, M. B. On the mechanism of the hydrogen–oxygen reaction on Pt(111). *Surf. Sci.* **416**, 37–58 (1998).

44. McEwen, J.-S., Gaspard, P., Visart de Bocarmé, T. & Kruse, N. Oscillations and Bistability in the Catalytic Formation of Water on Rhodium in High Electric Fields. *J. Phys. Chem. C* **113**, 17045–17058 (2009).

45. McEwen, J.-S., Gaspard, P., Visart de Bocarmé, T. & Kruse, N. Electric field induced oscillations in the catalytic water production on rhodium: A theoretical analysis. *Surf. Sci.* **604**, 1353–1368 (2010).

46. McEwen, J.-S., Gaspard, P., Visart de Bocarmé, T. & Kruse, N. Nanometric chemical clocks. *P. Natl. Acad. Sci. U.S.A.* **106**, 3006–3010 (2009).

47. Zeininger, J. *et al.* Reaction Modes on a Single Catalytic Particle: Nanoscale Imaging and Micro-Kinetic Modeling. *ACS Catal.* **12**, 12774–12785 (2022).

48. Winkler, P. *et al.* How the anisotropy of surface oxide formation influences the transient activity of a surface reaction. *Nat. Commun.* **12**, 69 (2021).

49. Kresse, G. & Furthmüller, J. Efficient iterative schemes for ab initio total-energy calculations using a plane-wave basis set. *Phys. Rev. B.* **54**, 11169 (1996).

50. Kresse, G. & Furthmüller, J. Efficiency of ab-initio total energy calculations for metals and semiconductors using a plane-wave basis set. *Comp. Mat. Sci.* **6**, 15–50 (1996).

51. Perdew, J. P., Burke, K. & Ernzerhof, M. Generalized gradient approximation made simple. *Phys. Rev. Lett.* **77**, 3865-8 (1996).

52. Blöchl, P. E. Projector augmented-wave method. *Phys. Rev. B.* **50**, 17953-79 (1994).

53. Blöchl, P. E., Först, C. J. & Schimpl, J. Projector augmented wave method: ab initio molecular dynamics with full wave functions. *Bull. Mat. Sci* **26**, 33–41 (2003).

54. Sharada, S. M., Karlsson, R. K., Maimaiti, Y., Voss, J., & Bligaard, T. Adsorption on transition metal surfaces: Transferability and accuracy of DFT using the ADS41 dataset. *Phys. Rev. B* **100**, 035439 (2019).

55. Bogojeski, M., Vogt-Maranto, L., Tuckerman, M. E., Müller, K. R., & Burke, K. Quantum chemical accuracy from density functional approximations via machine learning. *Nat. Commun.* **11**, 5223 (2020).56. Mavrikakis, M., Rempel, J., Greeley, J., Hansen, L. B., & Nørskov, J. K. Atomic and molecular adsorption on Rh(111). *J. Chem. Phys.* **117**, 6737–6744 (2002).

57. Ganduglia-Pirovano, M. V., & Scheffler, M. Structural and electronic properties of chemisorbed oxygen on Rh (111). *Phys. Rev. B* **59**, 15533 (1999).

58. Mavrikakis, M., Rempel, J., Greeley, J., Hansen, L. B., & Nørskov, J. K. Atomic and molecular adsorption on Rh (111). J. Chem. Phys. **117**, 6737–6744 (2021).
